# Population genetic analysis of the domestic Bactrian camel in China by RAD‐seq

**DOI:** 10.1002/ece3.5624

**Published:** 2019-09-26

**Authors:** Chenmiao Liu, Huiling Chen, Zhanjun Ren, Chengdong Zhang, Xuejiao Yang

**Affiliations:** ^1^ College of Animal Science and Technology Northwest A&F University Yangling China

**Keywords:** Bactrian camel, genetic relationship, RAD sequencing, single nucleotide polymorphisms

## Abstract

Restriction site‐associated DNA sequencing (RAD‐seq) is one of the most effective high‐throughput sequencing technologies for SNP development and utilization and has been applied to studying the origin and evolution of various species. The domestic Bactrian camels play an important role in economic trade and cultural construction. They are precious species resources and indispensable animals in China's agricultural production. Recently, the rapid development of modern transportation and agriculture, and the deterioration of the environment have led to a sharp decline in the number of camels. Although there have been some reports on the evolution history of the domestic Bactrian camel in China, the origin, evolutionary relationship, and genetic diversity of the camels are unclear due to the limitations of sample size and sequencing technology. Therefore, 47 samples of seven domestic Bactrian camel species from four regions (Inner Mongolia, Gansu, Qinghai, and Xinjiang) were prepared for RAD‐seq analysis to study the evolutionary relationship and genetic diversity. In addition, seven domestic Bactrian camel species are located in different ecological zones, forming different characteristics and having potential development value. A total of 6,487,849 SNPs were genotyped. On the one hand, the filtered SNP information was used to conduct polymorphism mapping construction, LD attenuation analysis, and nucleotide diversity analysis. The results showed that the number of SNPs in Dongjiang camel was the highest, the LD coefficient decayed the fastest, and the nucleotide diversity was the highest. It indicates that Dongjiang camel has the highest genetic diversity. On the other hand, the filtered SNPs information was used to construct the phylogenetic tree, and *F*_ST_ analysis, inbreeding coefficient analysis, principal component analysis, and population structure analysis were carried out. The results showed that Nanjiang camel and Beijiang camels grouped together, and the other five Bactrian camel populations gathered into another branch. It may be because the mountains in the northern part of Xinjiang and the desert in the middle isolate the two groups from the other five groups.

## Graphical abstract

In this paper, 47 samples of seven domestic Bactrian camel species from four regions (Inner Mongolia, Gansu, Qinghai, and Xinjiang) were prepared for RAD‐seq analysis to study the evolutionary relationship and genetic diversity. In addition, seven domestic Bactrian camel species are located in different ecological zones, forming different characteristics and having potential development value.

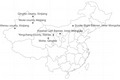

## 1 INTRODUCTION

The domestic Bactrian camel has been historically used as a transportation animal in the desert area of northwestern China and played an important role in ancient trade between China and Europe. Thus, the camel is very important for studying the livestock production in ancient northern China, and economic and cultural exchange between China and the West. With the rapid development of modern agriculture and transportation tools, the number of camels in China has dropped from 373,000 in 1994 to 250,000 today. Therefore, it is necessary to study the genetic diversity of Bactrian camels to clarify their phylogenetic relationship and lay the foundation for the protection of their biodiversity (Chen, Ren, Zhao, Zhang, & Yang, 2018). The genome of camel covers genetic information, which could be used to analyze the genetic relationship among different camels (He, Han, & Ma, 2009). The genetic diversity of six Bactrian camel populations in China was analyzed by microsatellite. It was found that Nanjiang camel and Beijiang camel gathered together, and the four groups of Alashan camel, Qinghai camel, Subei camel, and Sunite camel gathered together. Studies had shown that the genetic diversity of the domestic Bactrian camel in China was abundant and there was a certain gene flow among populations (Bai, Zhou, Li, & Feng, 2017). He (2011) studied the origin and domestication of nine domesticated Bactrian camel populations in China and one Mongolian domesticated Bactrian camel population by partial sequencing of mitochondrial D‐loop. Phylogenetic median‐joining network analysis showed that the domestic Bactrian camel populations came from a branch. However, the use of RAD‐seq for the development and utilization of genomic SNPs has not been reported in the genetic resources of Chinese Bactrian camel populations.

Owing to their high density and relatively uniform distribution throughout the genome, single nucleotide polymorphisms (SNPs) are ideal molecular markers for genetic mapping and population diversity assessment (Zhai et al., 2014). Currently, next‐generation sequencing (NGS) technologies have been used in phylogenetic analyses to quickly collect a wide range of molecular markers for elucidating evolutionary relationships (Zhou et al., 2018). The RAD‐seq has been proved to be economical and effective for understanding the evolutionary history within and between closely related species (Kang, Ma, & He, 2017). Jones, Fan, Franchini, Schartl, and Meyer (2013) used RAD‐Seq technology to analyze the phylogenetic relationship of 26 species from the Central American genus Xiphophorus, using a large number of SNP markers in the genome to infer the evolutionary relationship within the genus, improving the previous defects from the aspects of sequence and morphology. Zhai et al. (2014) used RAD‐seq technology in chickens for SNP discovery and genotyping, and found that RAD‐seq was a powerful tool for genotyping and discovering high‐density genetic markers. In addition, RAD‐seq can be used for deeper genetic analyses and applications, such as genome‐wide association studies (GWAS), marker‐assisted selection (MAS), and genome‐wide selection of other organisms.

In this study, RAD‐seq was used to analyze 47 samples of seven domestic Bactrian camel breeds from four regions (Inner Mongolia, Gansu, Qinghai, and Xinjiang) and to identify the SNPs of each sample. The study is beneficial to protect the genetic diversity and exploit the potential value of Bactrian camels. Camels are now more than a means of transportation. With the need of domestic animal products (camel wool, camel milk), especially the anticancer activity of camel milk (Krishnankutty et al., 2018), the genetic resources of Bactrian camels need to be redeveloped. In addition, seven kinds of domestic Bactrian camels located in different ecological zones have different characteristics and potential ecological adaptation value.

## 2 MATERIALS AND METHODS

### 2.1 Sample collection and DNA extraction

**Figure 1:**
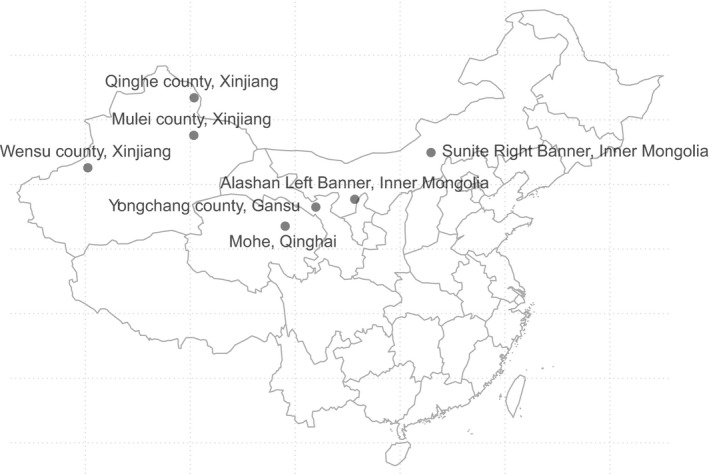
A geographical map of the sampling sites

In this study, 47 venous blood samples of 7 breeds of domestic Bactrian agricultural camel from Inner Mongolia, Gansu, Qinghai, and Xinjiang were collected (Table 1; Figure 1) to ensure that each sample came from a different family, and there was no relationship between individuals. The experiment was approved by the National Natural Science Foundation of China (31172178), and the relevant guidelines and regulations were followed. DNA samples were extracted using a standard phenol–chloroform method (Sambrook & Russell, 2002) and were diluted to 20 ng/µl.

**Table 1:** Sample information for domestic Bactrian camels in China

Number	Population	Code	Sampling site	Sample size
1	Nanjiang camel	NJ	Wensu County, Xinjiang	7
2	Beijiang camel	BJ	Qinghe County, Xinjiang	7
3	Dongjiang camel	DJ	Mulei County, Xinjiang	7
4	Hexi camel	HX	Yongchang County, Gansu	5
5	Qinghai camel	QH	Mohe, Qinghai	7
6	Alashan camel	ALS	Alashan Left Banner, Inner Mongolia	7
7	Sunite camel	SNT	Sunite Right Banner, Inner Mongolia	7
Total				47

### 2.2 Library construction

RAD‐seq libraries were constructed following a modified protocol (Baird et al., 2008). Briefly, genomic DNA (0.1–1 µg; from either individual or pooled samples) was digested with EcoRI (New England Biolabs) followed by heat inactivation of the enzyme. Individually barcoded P1 adapters were ligated onto the *EcoRI* cut site for each sample. Samples were pooled in proportionate amounts for shearing to average size of 500 bp. The library size was selected into 300‐ to 700‐bp fragments by running on a 1% agarose gel. Libraries were blunt‐end‐repaired, and a 3′‐adenine overhang was added to each fragment. We added a P2 adapter containing unique Illumina barcodes for each library. Libraries were amplified through 16 PCR cycles with Phusion high‐fidelity DNA polymerase (New England Biolabs) and column‐purified. Samples were sequenced on an Illumina HiSeq 3000 using 100 bp paired‐end reads.

### 2.3 Bioinformatics analysis

#### 2.3.1 Clean reads filtering

Quality trimming generated by the use of fastp is an essential step to generate high confidence of variant calling (Chen, Ren, et al., 2018; Chen, Zhou, Chen, & Gu, 2018). Raw reads would be processed to get high‐quality clean reads according to three stringent filtering standards: (a) removing reads with ≥10% unidentified nucleotides (N); (b) removing reads with >50% bases having phred quality scores of ≤20; and (c) removing reads aligned to the barcode adapter.

#### 2.3.2 SNP identification and annotation

To identify SNPs, the Burrows‐Wheeler Aligner (BWA) was used to align the clean reads from each sample against the reference genome (https://www.ncbi.nlm.nih.gov/genome/10741) with the settings “mem 4 ‐k 32 ‐M,” −k is the minimum seed length, and −M is an option used to mark shorter split alignment hits as secondary alignments (Li & Durbin, 2009). Variant calling was performed for all samples using the GATK's Unified Genotyper (Depristo et al., 2011). SNPs were filtered using the GATK's Variant Filtration with proper standards (‐Window 4, ‐filter “QD < 2.0||FS > 60.0||MQ < 40.0”,‐G_filter “GQ < 20”).

### 2.4 Statistical analysis

Statistically, comparing two of the multiple averages to each other is called multiple comparisons. There are many methods for multiple comparisons. In this study, multiple comparisons were made between SNPs of seven camel populations by using the least significant difference method (LSD method). The procedure of the least significant difference method is to calculate the least significant difference LSDα with a significant level of α under the premise that the *F* test is significant between the treatments. We used SPSS version 18.0 (SPSS, Inc.) for statistical analyses. The difference between any two averages, such as its absolute value ≥ LSD*α*, is significant at the level of *α*.

### 2.5 Population genetic analysis

The population relationship based on individual sample strategies was used to analyze the differentiation, gene exchange, and evolutionary history of seven domestic camel populations. Polymorphism map construction, LD attenuation analysis, and nucleotide diversity analysis were used for camel population genetic analysis.

#### 2.5.1 Polymorphism map construction

For each group, in order to ensure the reliability of the population SNP, for the sites where the SNP exists, each individual in the group needs to ensure that the site information exists, and missing phenomenon does not exist (Zhai et al., 2014). The vcftools software was used to calculate SNP statistics, the minimum read coverage for a SNP to be called is 3X, and all noncompletely missing polymorphic loci (–max‐missing 1e−06 –non‐ref‐af 1e−06) were used for counting. A total of 6,487,849 SNPs were obtained. Based on the information of each SNP locus, the unique SNP map was constructed for Chinese camel breeds.

#### 2.5.2 LD attenuation analysis

Linkage disequilibrium (LD) is also called as allelic association. It refers to a nonrandom association between different locus alleles within a population. After sequencing, 6,487,849 SNPs were obtained. We estimate the LD decay trend by calculating the LD coefficient (*r* ^2^) between two points in a range of sequence (typically < 5 Mb). The LD coefficient *r* ^2^ represents the correlation of two points, which varies within the interval of 0–1. If *r* ^2^ is 0, there is no correlation between the inheritance of the two locus. If *r* ^2^ is 1, it means that the site correlation is the largest. We used the “LD attenuation distance” to evaluate the speed of LD decay (Guo, 2012).

#### 2.5.3 Nucleotide diversity analysis

As an important indicator to measure the level of genetic variation of species, nucleotide diversity has important scientific significance for studying the genetic diversity of the species, its evolutionary history, and its systematic location. Nucleotide diversity can be calculated in the PopGenome package (Pfeifer, Wittelsbürger, Ramos‐Onsins, & Lercher, 2014). Nucleotide diversity analysis is a method for calculating the genetic diversity of individuals within a population by comparing the π value changes by using SNPs to calculate the average of the differences between any two nucleotide sequences in the population.

### 2.6 Population genetic relationship analysis

In the study, phylogenetic tree analysis, principal component analysis (PCA), population structure analysis, etc., were used to analyze the evolutionary relationship among different groups on different dimensions (Wang et al., 2015).

#### 2.6.1 Inbreeding coefficient analysis

The inbreeding coefficient refers to the correlation coefficient between two gametes forming an individual, that is, the ratio of the pair of genes from the common ancestor in all relative genes of the individual. Inbreeding coefficient was calculated using Plink 1.9 (http://zzz.bwh.harvard.edu/plink/). After *F* value of each individual was obtained, the average was obtained within the population.

#### 2.6.2 *F*_ST_ statistical analysis

Due to manual selection, there are significant differences between breeds in different regions. After removing the site with a deletion rate of 50% or more, the remaining 865,774 loci were used for the calculation of *F*_ST_. *F*_ST_ values can be calculated from the *F*_ST_ statistics function in the PopGenome package to study population genetic diversities among different breeds (Pfeifer et al., 2014). It can also help to study the genetic distance among different breeds and be used to express differences in population genetic structure.

#### 2.6.3 Phylogenetic tree analysis

The phylogenetic tree is a branch map or tree that describes the order of evolution between groups, indicating the evolutionary relationship between groups. After SNP detection, 6,487,849 SNPs can be used to calculate the distance between the populations. The phylogenetic tree was constructed by a neighbor‐joining method in the software Treebest (version 1.9.2), and a total of 1,000 replicates generated the Bootstrap values. Moreover, the phylogenetic tree was constructed by the maximum likelihood (ML) method and default parameters were set. By analyzing the phylogenetic tree constructed by the two methods, we can find the evolutionary relationship between populations.

#### 2.6.4 Principal component analysis

In genetics, PCA mainly converts multiple differences into a few independent factors and analyzes the differences among different groups by the degree of difference among these factors to classify different populations (Gao et al., 2017). In this study, after removing the site with a deletion rate of 50% or more, the remaining 865,774 loci were used for the calculation of PCA. We used the software GCTA (Yang, Lee, Goddard, & Visscher, 2011) for PCA analysis based on the degree of SNP differences between individuals, obtained the principal component values of each sample, and then used the R language to draw PCA scatter plots to further cluster different breeds.

#### 2.6.5 Group structure analysis

Genetic structure was inferred using the program STRUCTURE (http://web.stanford.edu/group/pritchardlab/structure.html; to ensure the independence of the SNP marks, we used the PLINK software to filter 865,774 SNPs according to the LD intensity, the remaining 12,046 loci were used for structure analysis, –indep‐pairwise 250 10 0.1, 250 kb window, the step size of 10 SNPs, *r* ^2^ is <.1). We predefined the number of genetic clusters from *K* = 2 to *K* = 6 (BURNIN = 5,000 times, NUMREPS = 100,000), and each *K* value was repeated three times. After the STRUCTURE software run was completed, web used the POPHELPER software (https://www.ncbi.nlm.nih.gov/pubmed/26850166) to calculate the ∆*K* value and the CLUMPP software (https://www.ncbi.nlm.nih.gov/pubmed/17485429) to merge the results of the three repetitions. After the combination, we used the POPHELPER software to illustrate it (Schraiber & Akey, 2015).

## 3 RESULTS

### 3.1 Sequencing basic data analysis

The ratio of high‐quality clean reads was above 97.36%, and the number of reads on the alignment was mostly above 97% by RAD‐seq. A total of 6,487,849 SNPs were detected. There were 2,270,747 loci (35%) in the transversion and 4,217,102 loci (65%) in the transition. The ratio of transition to transversion was nearly 2:1, the transition frequency between purine and pyrimidine was basically the same, and the transversion frequency of each type was also substantially flush.

### 3.2 Population genetic analysis

#### 3.2.1 Polymorphism map construction

**Figure 2:**
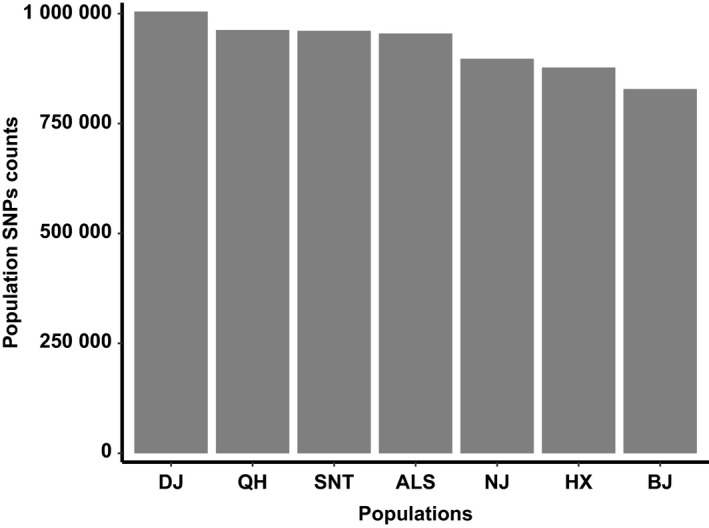
Population SNPs counts. The abscissa represents each population, and the ordinate represents number of SNPs per population. ALS, Alashan camels; BJ, Beijiang camels; DJ, Dongjiang camels; HX, Hexi camels; NJ, Nanjiang camels; QH, Qinghai camels; SNT, Sunite camels

The vcftools software was used to extract SNPs from each population, and there were differences in SNPs among different domestic camel populations. The number of SNPs in the seven domestic camel populations varied from 828,619 in the Beijiang camel to 1,004,857 in the Dongjiang camel, but the differences between the populations were not significant (*p* > .05; Figure 2). The differences of SNP in each population reflected the differences of polymorphism among different populations. The SNP map of the population was also constructed by the statistics of SNPs in different populations. It can be seen from the Figure 2 that the number of SNPs in Dongjiang camel was the highest, and the genetic diversity was highest.

#### 3.2.2 LD attenuation analysis

**Figure 3:**
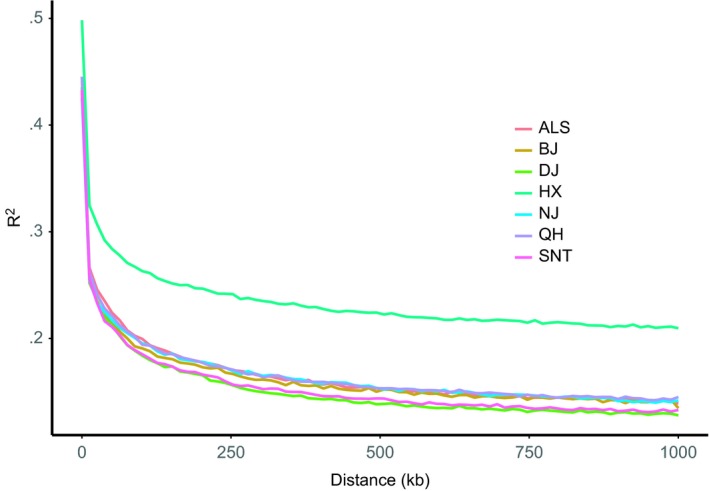
LD attenuation map of different camel populations. The abscissa represents the physical distance (bp), and the ordinate represents the LD coefficient (*R* ^2^)

The rate of LD attenuation varied greatly among different species or among different subgroups of the same species. The faster the LD coefficient decays, the higher the genetic diversity of the population is. LD attenuation analysis was performed on seven domesticated camel populations in four regions. It can be observed from the figure that the decay rate of the LD coefficient was different in different populations, and the attenuation speed was Dongjiang camel > Sunite camel > Beijiang camel > Nanjiang camel > Qinghai camel > Alashan camel > Hexi camel (Figure 3).

#### 3.2.3 Nucleotide diversity analysis

Nucleotide diversity represents the genetic diversity of population to some extent. It can be seen from Table 2 that the Dongjiang camel had the highest nucleotide diversity, and the genetic diversity was highest. The nucleotide diversity of Hexi camel was low. It is consistent with the results of polymorphism map construction and LD attenuation analysis.

**Table 2:** *F*_ST_, *π*, and *F*_IS_ values for domestic Bactrian camels in China

Population	Code	Average_*F*_ST_	*π*	Average_*F*_IS_
Nanjiang camel	NJ	0.1971	0.0001516	0.4515
Beijiang camel	BJ	0.1991	0.0001368	0.4947
Dongjiang camel	DJ	0.1594	0.0001716	0.3959
Hexi camel	HX	0.2068	0.0001489	0.4193
Qinghai camel	QH	0.2020	0.0001573	0.3782
Alashan camel	ALS	0.1834	0.0001625	0.4361
Sunite camel	SNT	0.1742	0.0001627	0.4507

### 3.3 Population genetic relationship analysis

#### 3.3.1 Inbreeding coefficient analysis

For an individual, the probability of getting a gene from the parent is 1/2, and the probability of getting a gene from the grandparent is 1/4; that is, the probability of getting the same gene from a common ancestor is reduced by 1/2 every other generation. Therefore, the closer the kinship is, the greater the inbreeding coefficient is. As can be seen from Table 2, the average value of *F*_IS_ between Hexi camel, Qinghai camel, and other populations was lower, indicating that the genetic distance was far from other populations.

#### 3.3.2 *F*_ST_ statistical analysis

The *F*_ST_ values between different populations were calculated to study the genetic diversity among different populations. It can be seen from Table 2 that the average *F*_ST_ value between Dongjiang camel and other camel breeds was 0.1594, which was the lowest among all the average values. It indicated that its genetic distance was close to other populations, and the genetic difference with other populations was not significant. The average value of *F*_ST_ between Hexi camel, Qinghai camel, and other populations was the highest, indicating that the genetic distance was far from other populations.

#### 3.3.3 Phylogenetic tree analysis

**Figure 4:**
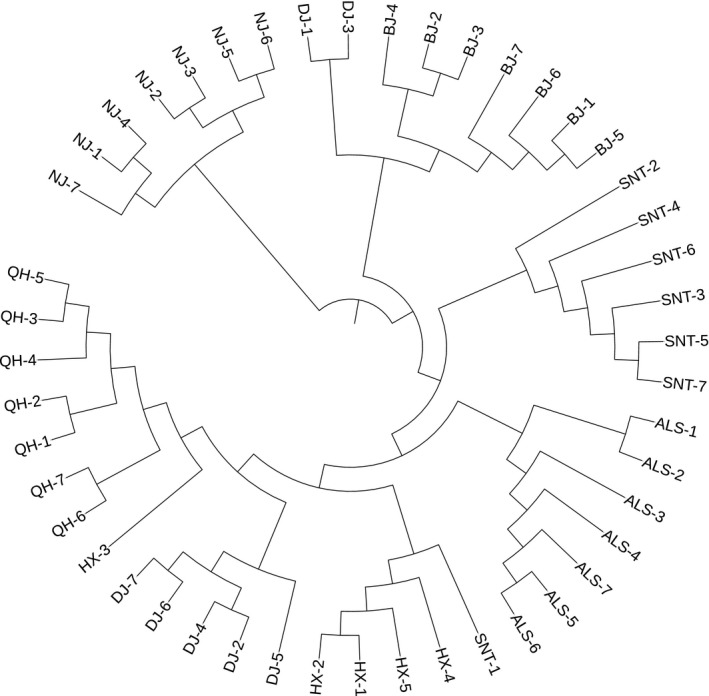
Phylogenetic tree construction by maximum likelihood method. ALS, Alashan camels; BJ, Beijiang camels; DJ, Dongjiang camels; HX, Hexi camels; NJ, Nanjiang camels; QH, Qinghai camels; SNT, Sunite camels

**Figure 5:**
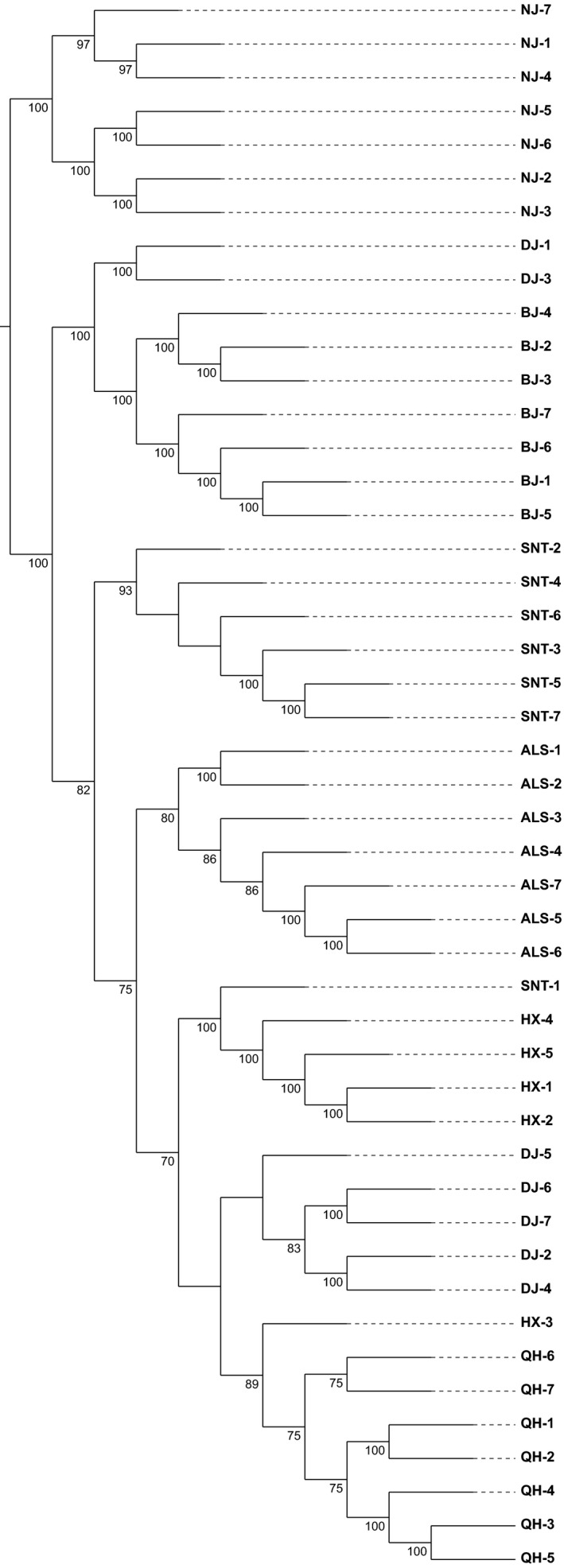
Phylogenetic tree construction of neighbor‐joining (NJ) method. ALS, Alashan camels; BJ, Beijiang camels; DJ, Dongjiang camels; HX, Hexi camels; NJ, Nanjiang camels; QH, Qinghai camels; SNT, Sunite camels

The filtered SNPs information was used to construct phylogenetic tree by the ML method (Figure 4) and the NJ method (Figure 5). The results showed that the seven breeds were clustered together, and the clusters were obvious, and the order of evolution was consistent. Two populations of Beijiang camel and Nanjiang camel were closely related and can be regarded as a large branch. Five populations of Qinghai camel, Dongjiang camel, Hexi camel, Alashan camel, and Sunite camel can be regarded as another branch. Some individuals of Dongjiang camel and Beijiang camel formed branches. It indicated that there may be gene communication between Beijiang camel and Dongjiang camel.

#### 3.3.4 Principal component analysis

**Figure 6:**
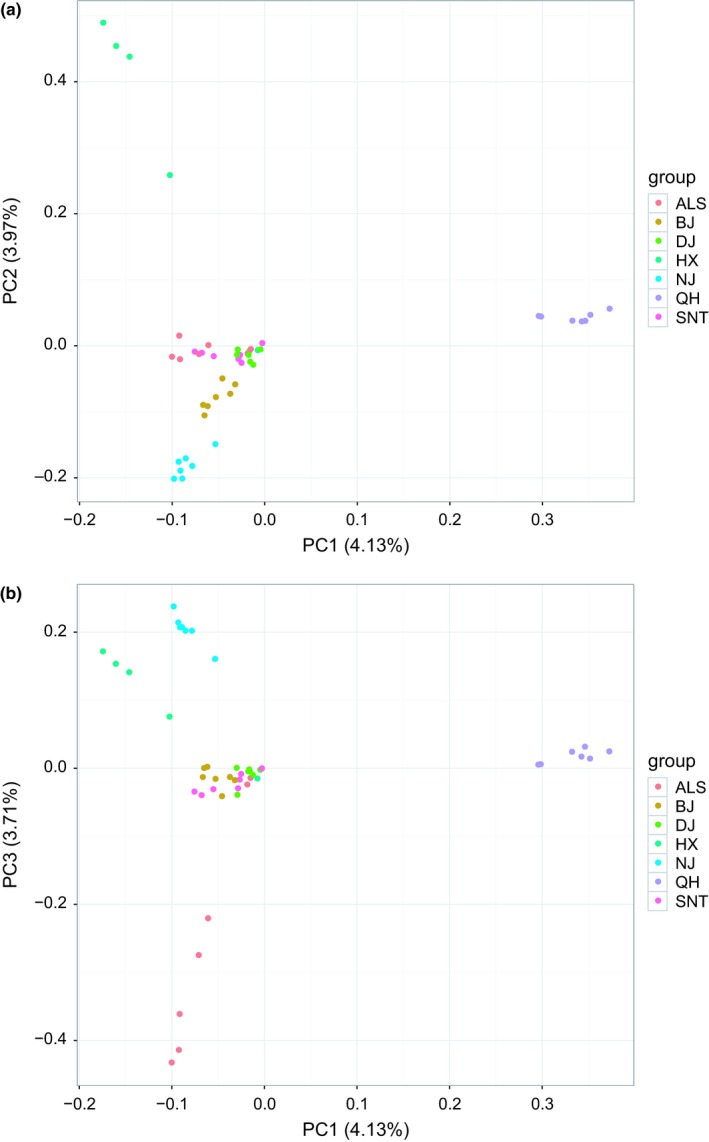
Principal components analysis in domestic Bactrian camel populations (1) and (2)

The key information was extracted from millions of SNPs, and the PCA of seven breeds of domestic camels was conducted (Figure 6). On the PC1 and PC2 level, Qinghai camels were grouped together, and Hexi camels were grouped together and were far away from other groups. It was consistent with the *F*_ST_ statistical analysis. Two PCA maps showed that Sunite camel and Alashan camels were clustered together, and there was genetic communication between two camel breeds. They had a close genetic relationship, and it was consistent with the results of the phylogenetic tree. Dongjiang camels and Beijiang camels gathered together, that showed genetic communication and relatively close genetic relationship. It was consistent with the results of the phylogenetic tree.

#### 3.3.5 Group structure analysis

**Figure 7:**
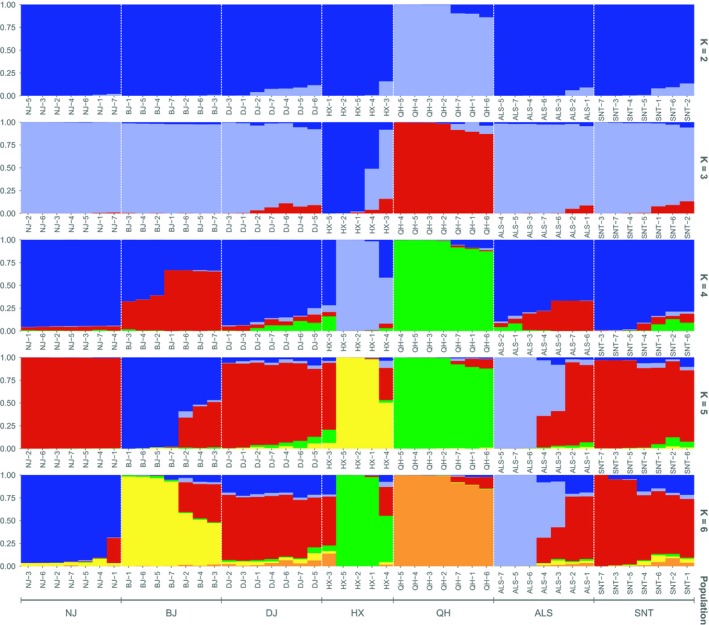
Groups structure clustering figure in domestic Bactrian camel populations. ALS, Alashan camels; BJ, Beijiang camels; DJ, Dongjiang camels; HX, Hexi camels; NJ, Nanjiang camels; QH, Qinghai camels; SNT, Sunite camels

The STRUCTURE analysis software was used to analyze the population structure of the domestic Bactrian camels and the ancestral population of each breed (Figure 7). When performing a population structure analysis for each *K* value, we performed cross‐validation to determine which *K* value best fitted the true differentiation history of the population. In Figure 7, the best classification strategy for this group was *K* = 5. At *K* = 3, the Qinghai camel and the Hexi camel were clearly separated from other populations of camels, indicating that the Qinghai camel and the Hexi camel were far away from the camels of other populations, which was consistent with the results of the *F*_ST_ statistical analysis. At *K* = 5, the Alashan camel and the Sunite camel shared a common genetic ancestry, and there was genetic communication between two populations of camels, which was consistent with the results of the PCA. Some individuals of the Dongjiang camel shared an ancestral genetic background with the Beijiang camel, which was consistent with the results of the evolutionary tree. The ancestral background of Nanjiang camel was very pure and had a genetic ancestry. Dongjiang camel, Qinghai camel, and Sunite camel had a variety of genetic ancestry, but there was a main genetic ancestry.

## 4 DISCUSSION

The genetic diversities and evolutionary origins of domestic Bactrian camels in China have been a fascinating source for decades (Chen, Ren, et al., 2018; Chen, Zhou, et al., 2018). We used RAD‐seq technology to analyze the SNP loci of domestic Bactrian camels and further carried out the population genetic research of domestic Bactrian camels. A total of 6,487,849 SNP loci were detected in all samples. Filtered SNPs were conducted for polymorphism map construction, LD decay analysis, and nucleotide diversity analysis. Additionally, the phylogenetic tree analysis, *F*_ST_ statistical analysis, inbreeding coefficient analysis, principal component analysis, and population structure analysis were employed to analyze the population genetic relationship. The results showed that the number of SNPs in Dongjiang camel was the highest, the LD coefficient decayed the fastest, the nucleotide diversity was the highest, and the genetic diversity was the highest, which may be due to the preservation of genetic diversity of ancestors (Abdulla et al., 2009). From the PCA map and the phylogenetic tree map, we can see that the Dongjiang camels clustered in two different branches. It indicates that Dongjiang camel may be mixed with other populations of Bactrian camels, resulting in higher genetic diversity of Dongjiang camel. In addition, the seven breeds of domesticated Bactrian camels were distinctly grouped and there was extensive genetic communication between the breeds. Nanjiang camel and Beijiang camel gathered together, and the other five Bactrian camel groups (including Dongjiang camel, Hexi camel, Qinghai camel, Alashan camel, and Sunite camel) gathered together. It is consistent with the existing literature (Bai et al., 2017; Wang, Ren, Chen, Wang, & Wang, 2010). Since some previous studies on Chinese Bactrian camels (Gao, Wang, He, Chen, & Meng, 2009; Han, Ochieng, Lkhagva, & Hanotte, 2004) did not involve Xinjiang groups, the selected groups were all in the second branch of this study, so it was concluded that there was no obvious genetic differentiation in the domestic Bactrian camel in China (He et al., 2012). Geographically, the Altai Mountains in the northern part of Xinjiang, the Tianshan Mountains, and the Taklimakan Desert in central Xinjiang have formed a natural barrier that isolates the Nanjiang camel and the Beijiang camel from the other five groups. In another branch, the Sunite camel had a far‐reaching genetic relationship with other groups, which may be related to its geographical distribution. In the south of Xilin Gol League, where the Sunite camel is distributed, the Yinshan Mountains form a natural barrier that blocks communication between the group and other groups (Bai et al., 2017). The Alashan camel and the Sunite camel had genetic communication, and the genetic relationship was relatively close. This may be related to the fact that they belong to the Inner Mongolia camel and are geographically close. In the phylogenetic tree analysis, the genetic relationship among Qinghai camel, Hexi camel, and Alashan camel was relatively close, which may be related to its geographical distribution. They are all distributed near Qilian Mountain.

In the study, RAD‐seq was first applied on camels. RAD‐seq technology includes DNA sequence data from multiple loci in the genome at a lower cost, simpler library preparation process, no prior knowledge of any locus DNA sequences, management of data from simplified genomes, and well‐developed pipelines for data treatment and analysis to retrieve orthologous sites for comparison (Eaton & Ree, 2013; McCormack, Hird, Zellmer, Carstens, & Brumfield, 2013; Rubin, Ree, & Moreau, 2012; Zhou et al., 2018). So far, various studies have been carried out on the origin, domestication, and genetic evolution of camels at home and abroad. Zhang (2014) studied the mitochondrial Cytb gene and D‐loop sequence of the domestic Bactrian camel in China. Phylogenetic tree and network analysis indicated that all domestic Bactrian camels were clustered into one group and belonged to a single maternal origin. The mitochondrial genome on large‐scale, wide‐spread, domesticated Bactrian camels (including Mongolia, Russia, and China) was studied. It indicated that most of the mitochondrial haplotypes of domestic Bactrian camels were shared, supporting the claim that the domestic Bactrian camels belonged to the single maternal origin (Ming et al., 2017). At present, the earliest evidence for the domestication of Bactrian camels is concentrated in the northeast of Ilang and the neighboring southern Turkmenistan, especially in the Kopet Daghmountain region. Bactrian camel bones were found in the second cultural layer of the Anau site on the northern edge of the mountain range, dating back to 3500–3000 BC (Meadow & Zeder, 1978). However, the earliest archeological evidence of domestic Bactrian camels in China was the buck cemetery of Luntai group, which can be estimated as early as 800BC. Combined with the materials of the similar Nomhong ruins, it can be clearly assumed that there were domestic Bactrian camels in the northwestern part of China in the late Western Zhou Dynasty (Zhang & Luo, 2014). Domestic Bactrian camels are less likely to be domesticated in China and are more likely to be introduced directly from abroad (Bulliet, 1975). The most likely route is starting at Turkmenistan, passing successively through the south coast of the Aral Sea, today's Kazakhstan, Kyrgyzstan, the north and south of the Tianshan Mountains into the northern Xinjiang, and then getting to the Hexi corridor (Zhang & Luo, 2014). In addition, most experts have devoted themselves to the study of genomic DNA of Bactrian camels using microsatellites to reveal the genetic evolution of Bactrian camels distributed in different regions, providing basic research materials for the protection and utilization of Bactrian camels (Gao et al., 2017).

Male‐specific region of Y chromosome in mammals does not recombine with X chromosome during meiosis. It has low mutation rate and is easy to form specific haploid. It follows strict paternal inheritance, so it is an important genetic resource for studying paternal genetic diversity (Cao et al., 2018). Chen, Ren, et al. (2018) and Chen, Zhou, et al. (2018) studied single nucleotide polymorphisms, microsatellites, and copy number variation (CNV) on the Y chromosome of Chinese domesticated Bactrian camels to understand Y chromosome polymorphism of Chinese domesticated Bactrian camels. The polymorphic markers of the male‐specific portion of the Y chromosome (MSY) provide useful information for tracking male genealogies. Based on single nucleotide variants, a Y‐phylogenetic network with seven haplotypes found that wild and domestic Bactrian camels were obviously divided into two distinct groups (Felkel et al., 2019). Therefore, we need high‐throughput sequencing to analyze the camel's Y chromosome to discover polymorphic markers on the Y chromosome in the next step, further enriching our results. In addition, we will actively seek cooperation with foreign countries in the next step of research, expanding camel blood samples along the Silk Road to further reveal the phylogenetic relationship of camels.

## CONFLICT OF INTEREST

None declared.

## AUTHOR CONTRIBUTIONS

Zhanjun Ren and Chenmiao Liu designed the study and wrote the manuscript, Huiling Chen collected the samples, and Chengdong Zhang and Xuejiao Yang contributed to the data analysis. All authors read and approved the manuscript.

## Data Availability

Domestic Bactrian camel DNA sequences: All raw RAD‐seq reads data can be accessed at NCBI SRA. Bioproject # PRJNA522647, Biosample #s SAMN10948548–SAMN10948594.
